# A Zinpyr-1-based Fluorimetric Microassay for Free Zinc in Human Serum

**DOI:** 10.3390/ijms20164006

**Published:** 2019-08-16

**Authors:** Wiebke Alker, Tanja Schwerdtle, Lutz Schomburg, Hajo Haase

**Affiliations:** 1 Department of Food Chemistry and Toxicology, Berlin Institute of Technology, 13355 Berlin, Germany; 2 TraceAge-DFG Research Unit on Interactions of Essential Trace Elements in Healthy and Diseased Elderly, Potsdam-Berlin-Jena, Germany; 3 Institute of Nutritional Science, University of Potsdam, Arthur-Scheunert-Allee 114–116, 14558 Nuthetal, Germany; 4 Institute for Experimental Endocrinology, Charité-Universitätsmedizin Berlin, corporate member of Freie Universität Berlin, Humboldt-Universität zu Berlin, and Berlin Institute of Health, Augustenburger Platz 1, 13353 Berlin, Germany

**Keywords:** zinc, free zinc, serum, biomarker, fluorescent probe, Zinypr-1

## Abstract

Zinc is an essential trace element, making it crucial to have a reliable biomarker for evaluating an individual’s zinc status. The total serum zinc concentration, which is presently the most commonly used biomarker, is not ideal for this purpose, but a superior alternative is still missing. The free zinc concentration, which describes the fraction of zinc that is only loosely bound and easily exchangeable, has been proposed for this purpose, as it reflects the highly bioavailable part of serum zinc. This report presents a fluorescence-based method for determining the free zinc concentration in human serum samples, using the fluorescent probe Zinpyr-1. The assay has been applied on 154 commercially obtained human serum samples. Measured free zinc concentrations ranged from 0.09 to 0.42 nM with a mean of 0.22 ± 0.05 nM. It did not correlate with age or the total serum concentrations of zinc, manganese, iron or selenium. A negative correlation between the concentration of free zinc and total copper has been seen for sera from females. In addition, the free zinc concentration in sera from females (0.21 ± 0.05 nM) was significantly lower than in males (0.23 ± 0.06 nM). The assay uses a sample volume of less than 10 µL, is rapid and cost-effective and allows us to address questions regarding factors influencing the free serum zinc concentration, its connection with the body’s zinc status, and its suitability as a future biomarker for an individual’s zinc status.

## 1. Introduction

Zinc is an essential trace element with crucial functions for a myriad of biological processes [1,2,3]. It is estimated that approximately 20% of the global population is at risk of zinc deficiency [4]. This undersupply can affect processes such as growth, reproduction, neurological functions and the immune response [3,5]. The total concentration of zinc in serum or plasma is a commonly used biomarker for zinc deficiency. This parameter is well suited for identifying the zinc status of a population. However, it has its limitations when it comes to an individual’s zinc status. It shows a lack of sensitivity towards moderate zinc restriction, as it took highly undersupplied conditions to cause a decline in the total serum zinc concentration, whereas a moderate undersupply did not have an appreciable effect [6]. Also, inter-individual differences regarding the magnitude and the velocity of alterations in serum zinc concentration due to a restriction have been observed [6]. Hence, a suitable biomarker for the zinc status of an individual that responds well to marginal forms of limited zinc availability is still missing [6].

In human serum 80–90% of zinc is bound to albumin and 10–20% to alpha-2-macroglobulin [7]. Variations in the total serum zinc concentration were shown to correlate with albumin-bound zinc, whereas no correlation between the fraction of zinc bound to alpha-2-macroglobulin with total serum zinc has been observed [8,9]. In addition, there is also a very small pool of zinc that is not tightly bound to proteins, but relatively weakly bound to low molecular weight fractions and/or rapidly exchanges ligands [10,11]. This pool is referred to as free zinc or by several synonyms including “labile”, “bioavailable” and “rapidly exchangeable” zinc. Regarding the issue of free zinc, it has to be noted that it lacks a clear definition, so far, and might be defined differently by various authors. For example, Zalewski et al. include the albumin-bound fraction of zinc when referring to “labile pools of zinc” [12], which differs from the aforementioned definition of free zinc that will be used in the present manuscript.

Several studies in cell culture suggest that the concentration of free zinc, rather than the total amount of zinc in the medium, is decisive for aspects such as toxicity of zinc or zinc-induced cytokine production [13,14,15]. This zinc pool seems to be the one that is actually biologically active and available to interact with cells. For this reason, the free zinc concentration in the serum might be a better parameter than total serum zinc to reflect the body’s zinc status and might serve as a suitable biomarker for individuals [16].

The present study aimed to develop a method for determining free zinc in human serum based on the fluorescent probe Zinpyr-1. In many clinical cohorts available sample volumes are strictly limited, and because of this the assay should be feasible with a maximum of 10 µL serum per patient. The parameters for zinc quantification were optimized regarding concentrations of the assay components and incubation times. As a first test, the method was applied to a panel of 154 human serum samples, measuring free zinc and analyzing its correlation with the total concentrations of zinc, manganese, iron, copper or selenium and factors such as age or gender.

## 2. Results and Discussion

### 2.1. Excitation and Emission of Zinpyr-1

**Figure 1:**
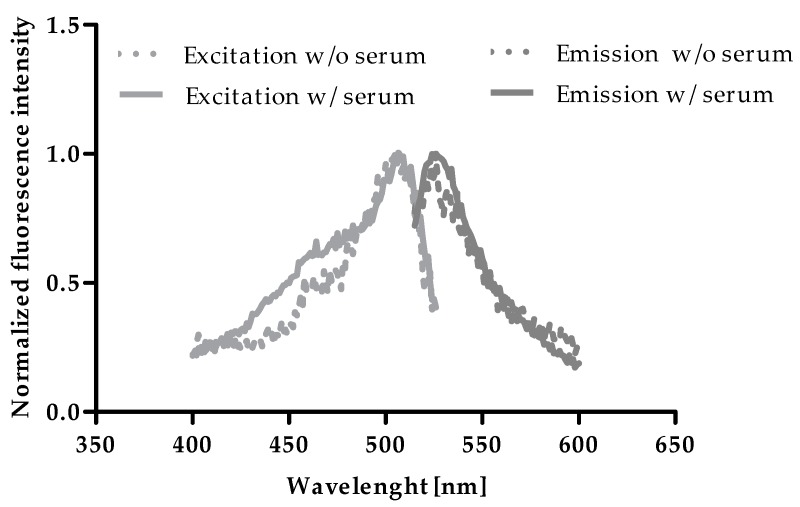
Excitation and emission of Zn^2+^-saturated Zinpyr-1 in the presence and absence of human serum. Excitation (400–526 nm) and emission (515–600 nm) spectra were acquired from Zinpyr-1 [0.05 µM] in the presence of ZnSO_4_ [10 mM] in assay-buffer with and without addition of 2% (v/v) of HS.

To test a potential influence of serum on excitation and emission maxima of Zinpyr-1, excitation and emission scans of zinc-saturated Zinpyr-1 were compared in the absence and presence of HS. As shown in Figure 1, maxima were not affected by the addition of HS and were determined at 507 nm and 526 nm, respectively, which is in line with values in the literature [17,18].

### 2.2. Optimization of Incubation Time for F

**Figure 2:**
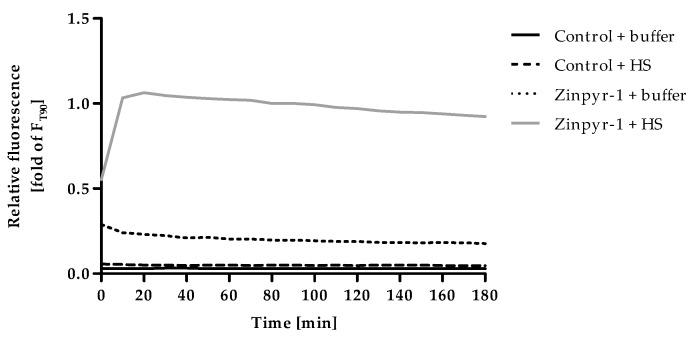
Kinetics of F. Controls (assay-buffer alone) or Zinpyr-1 (final concentration 0.05 µM in assay-buffer) were equilibrated to 37°C and the experiment started by adding 2% (v/v) human serum (HS) or an equal volume of assay-buffer. Fluorescence was measured in 10 min intervals for a total duration of 180 min. Data are shown relative to the fluorescence of Zinpyr-1 + HS after 90 min (= value for F used in the final assay procedure) and represent means of n = 3 independent experiments.

Time-resolved measurements were conducted to follow the changes of Zinpyr-1 fluorescence after addition of HS (Figure 2). In addition, the fluorescence intensity of the assay-buffer, alone and supplemented with either human serum or Zinpyr-1 was recorded. The autofluorescence of the buffer with and without serum was negligible, while Zinpyr-1 showed some basal fluorescence that slightly but continuously decreased throughout the measurement. Addition of HS to Zinpyr-1 resulted in a steep increase of fluorescence within the first minutes reaching a gradually descending plateau. Even though this decrease is only a minor effect, the time at which the measurement of F is performed should be kept constant to ensure maximum reproducibility. To allow sufficient time for formation of the equilibrium between zinc, serum components and the fluorescent dye, the incubation time for F was set at 90 min in all subsequent experiments.

### 2.3. Optimization of Incubation Time, Chelator Type and Concentration for F_min_

**Figure 3:**
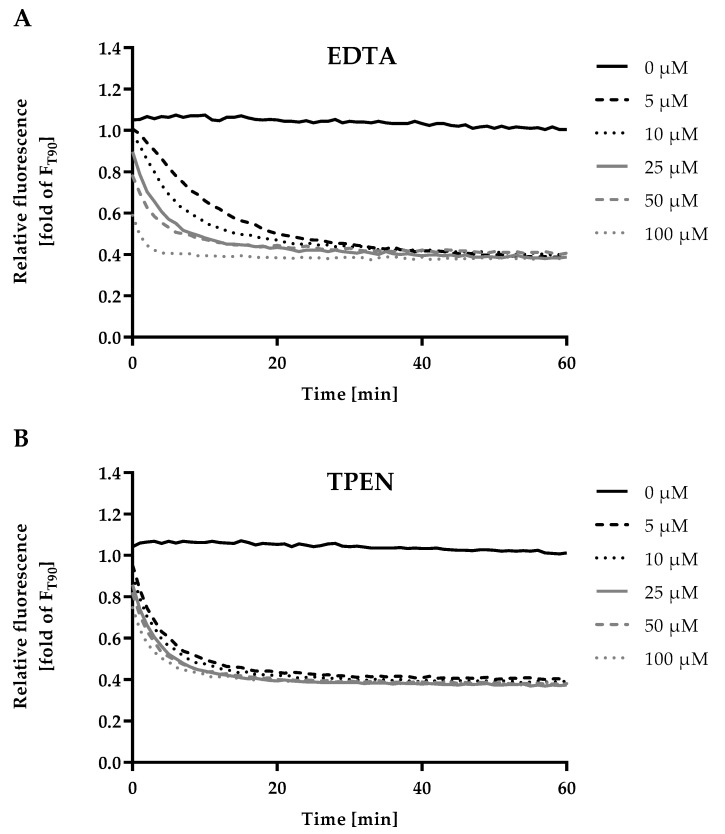
Kinetics of F_min_. The final assay procedure was performed up to the measurement of F as described in Materials and Methods and the experiment was started by addition of either (**A**) ethylenediaminetetraacetic acid (EDTA) or (**B**) N,N,N′,N′-tetrakis(2-pyridinylmethyl)-1,2-ethanediamine (TPEN) solution (final concentrations as indicated). Fluorescence was measured in 1 min intervals for a total duration of 60 min. Data are shown relative to the value for F used in the final assay procedure, representing means of at least n = 3 independent experiments.

EDTA as well as TPEN chelate zinc and can be used to determine F_min_ by withdrawing zinc ions from the fluorescent probe [19]. Figure 3 shows a concentration-dependent decline of the fluorescence intensity after addition of the chelators EDTA (Figure 3A) or TPEN (Figure 3B). Both chelators yield comparable minimal fluorescence signals, given that sufficient concentrations and incubation times are met, and could therefore be applied in the assay. For subsequent experiments 100 µM EDTA and an incubation time of 20 min were chosen to induce F_min_. 

### 2.4. Optimization of Incubation Time and Zinc Concentration for F_max_

**Figure 4:**
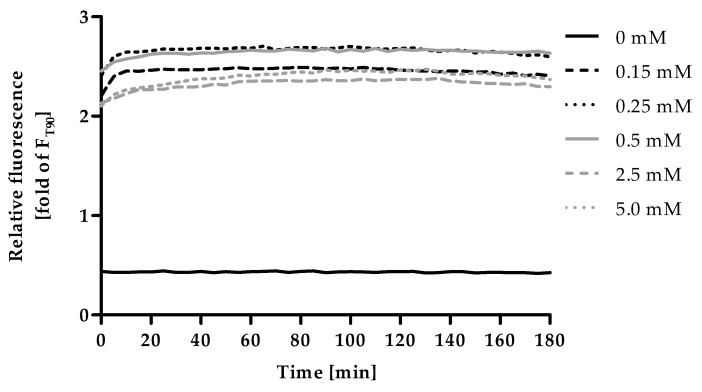
Kinetics of F_max_. The final assay procedure was performed up to the measurement of F_min_ as described in Materials and Methods and the experiment was started by the addition of different ZnSO_4_-solutions (final concentrations as indicated). Fluorescence was measured in 5 min intervals for a total duration of 180 min. Data are shown relative to the value for F used in the final assay procedure, representing means of n = 3 independent experiments.

Excess ZnSO_4_ was applied to saturate the fluorescent probe with zinc in order to induce F_max_. Within minutes the addition of ZnSO_4_ led to a strong increase in fluorescence compared to the control, (Figure 4), which was stable throughout the experimental duration of 180 min. The fluorescence intensity of 0.15 mM ZnSO_4_ is lower compared to 0.25 mM and 0.5 mM, indicating that this concentration is not yet sufficient to saturate the probe completely. The application of 0.25 mM and 0.5 mM yield the highest fluorescence intensity. Even higher concentrations (2.5 mM and 5 mM) resulted in a decrease of the fluorescence intensity. This is most likely due to the formation of a white precipitate, which was observed in the respective wells after addition of zinc. Consequently, for subsequent experiments, 0.5 mM ZnSO_4_ and an incubation time of 90 min were chosen for measurement of F_max_.

### 2.5. Zinpyr-1 Concentration 

**Figure 5:**
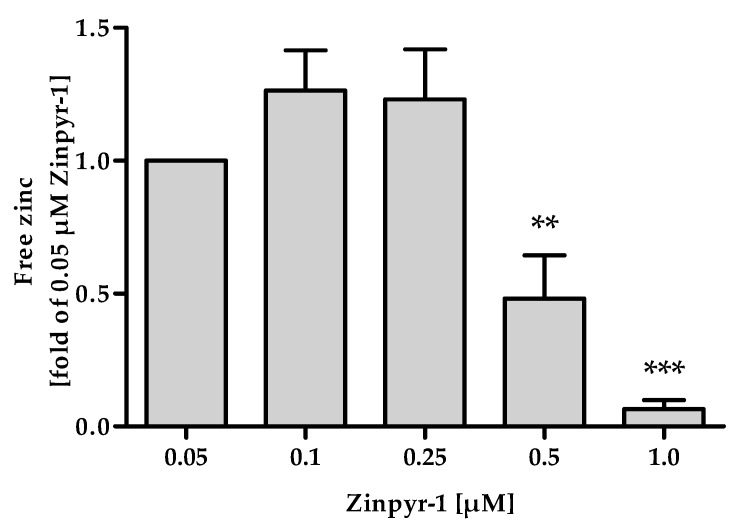
Effect of Zinpyr-1 concentration on free zinc measurements. The final assay procedure was performed as described in Materials and Methods, using the indicated concentrations of Zinpyr-1. Data are shown as means ± SD of n = 3 independent experiments. Significant differences to the mean measured for 0.05 µM Zinpyr-1 are indicated (** *p* < 0.01, *** *p* < 0.001; one-way analysis of variance (ANOVA) with Bonferroni post hoc test).

The applied concentration of Zinpyr-1 needs to be as low as possible, because the addition of a probe that acts by chelating its analyte influences the equilibrium between free and protein-bound zinc. If the concentration of the fluorescent probe is too high, its fractional saturation is no longer determined by its affinity, but limited by the availability of zinc, leading to underestimation of the free zinc concentration [20]. This can be seen in Figure 5, where concentrations of 0.5 µM and above yield significantly reduced values for the free zinc concentration.

### 2.6. Saturation of the Fluorescent Probe

**Figure 6:**
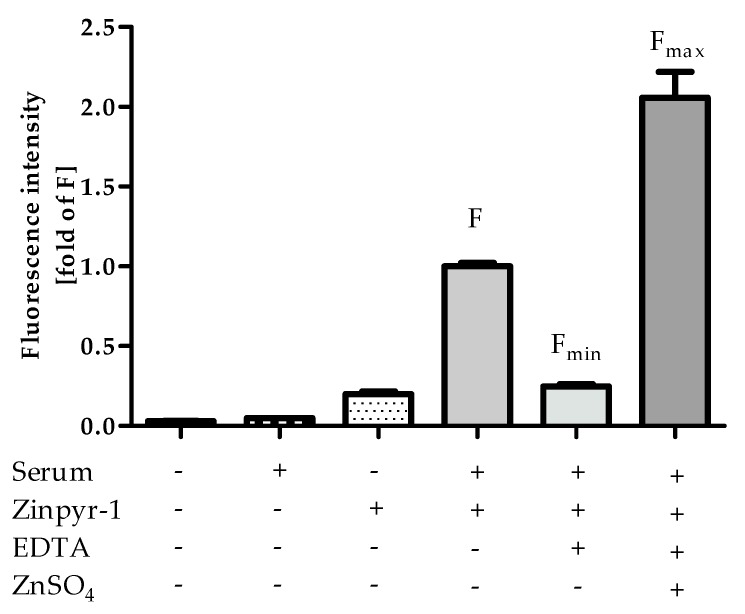
Comparison of fluorescence intensities of controls F, F_min_ and F_max_. Values were extracted from Figure 2, Figure 3A and Figure 4 are shown relative to F. Data represent means ± SD from n = 3 independent experiments.

Ideally, the affinity of a probe should match the actual concentrations in typical samples, allowing precise determination and leaving room for sera with higher and lower free zinc concentrations. As it can be seen in Figure 6, the saturation of Zinpyr-1 with zinc ions from HS, indicated by the relation of F_max_ to F, is roughly in the middle of its dynamic range. Comparable experiments were also performed with FluoZin-3 (data not shown), which has a higher K_D_ of 8.9 nM [21]. Accordingly, only a small fraction of the lower-affinity probe FluoZin-3 was in its zinc-bound state after addition of HS, making its affinity less well suited for analyzing free zinc in human sera compared to Zinpyr-1, in particular for samples with lower free zinc content.

One additional piece of information that can be gained from Figure 6 is that there is no notable difference between the fluorescence of Zinpyr-1 in the absence of HS and F_min_. This confirms that the assay buffer and Zinpyr-1 are of sufficient purity and do not contribute measurable amounts of free Zn to the experimental system, leaving the investigated sera as the only source for Zn.

### 2.7. Determination of Free Zinc in Human Serum Samples

The established method was applied to 154 commercially obtained sera from healthy human subjects. The measured average free zinc concentration was 0.22 ± 0.05 nM, ranging between 0.09 and 0.42 nM. Since the 1980s, different approaches have been applied in order to measure the concentration of non-protein-bound or free zinc in serum samples. Using ultrafiltration two studies determined concentrations of non-protein-bound zinc of 81 ± 16 nM and 27 ± 3 nM, respectively [22,23]. This is higher than the results from our method, but includes zinc bound by low molecular weight ligands in addition to free zinc. Using phosphoglucomutase as a metal ion indicator, a free zinc concentration of 0.2 nM in equine plasma has been measured [24] and dialysis of bovine plasma yielded a free zinc concentration of 0.141 nM [25]. Moreover, the fluorescent probes ZnAF-2 and Zinpyr-1 have been used to measure free zinc in rat plasma and porcine serum, finding concentrations of 1 nM [26] and 0.1 nM [27], respectively. Our values from human sera are in good agreement with these data.

**Figure 7:**
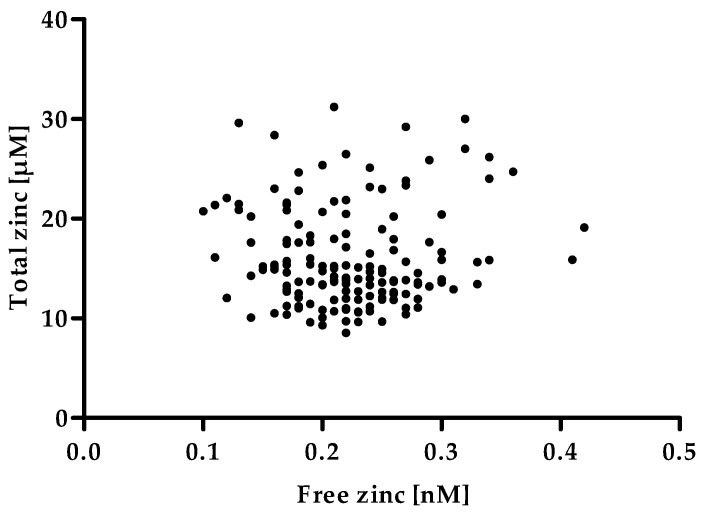
Correlation between free zinc and total zinc concentrations in human serum samples. The total zinc concentration was measured by inductively coupled plasma mass spectrometry (ICP-MS). Data represent values of a single determination. Free zinc concentration was measured using the method established above and represent means of n = 3 independent experiments. No significant correlation was found between total and free zinc concentration of the serum samples (Spearman rank correlation, α = 0.05).

As depicted in Figure 7 the free zinc concentration of the serum samples does not correlate with the total zinc concentration. Such a correlation would have severely limited the usefulness of measuring the serum free zinc concentration as a biomarker for zinc status, as it would not have offered a remarkable gain in information in addition to the total serum zinc concentration. Consequently, the free zinc concentration would most likely have shown the same drawbacks as a biomarker for an individual’s zinc status, such as the lack of sensitivity toward moderate zinc undersupply [6]. However, the absence of a correlation supports the meaning of the serum free zinc concentration as an independent parameter besides the total serum zinc concentration. As it has been suggested that free zinc represents the biologically available zinc [13,14,15], this parameter could be of high physiological relevance, making it promising to further investigate it as a possible biomarker.

Perspectively, factors influencing the free serum zinc concentration have to be identified; some can already be found in the literature. Serum zinc is mainly bound to proteins such as albumin or alpha-2-macroglobulin; therefore their binding capacity is one factor most likely influencing the free zinc concentration. In addition to proteins, serum contains various nutrients and metabolites. Many of them are bound to transport proteins while in the serum or exist in different species. Notably, the concentration of one compound can influence the species in which another compound is present. For albumin, an impact of fatty acids on its zinc-binding ability has been shown. The major zinc binding site of albumin (site A) experiences a conformational change due to fatty acid binding to the fatty acid binding site 2 (FA2), making simultaneous binding of zinc and fatty acids to albumin incompatible. Therefore, the fatty acid concentration seems to influence zinc binding by albumin and the free zinc concentration in the serum [28].

Amino acids might influence zinc speciation in the serum, as well. In 1970, Prasad and Oberleas observed an increase of ultrafiltrable zinc after the addition of physiological concentrations of amino acids to pre-dialyzed human serum. Histidine, glutamine, threonine, cysteine and lysine had the largest effect. It was shown that zinc is bound to a number of different proteins, among which the amino acids competed with albumin, haptoglobin, transferrin and immunoglobulin G for zinc, but not with ceruloplasmin and alpha-2-macroglobulin [10].

Additionally the body has different mechanisms to alter the concentration of certain compounds to adjust to different situations. Factors such as infections or stress cause alterations in zinc homeostasis. In a porcine model of sepsis a decrease in the total and free serum zinc concentrations preceded the decline of total serum protein levels, and was accompanied by a loss in the zinc binding capacity of the sera [27]. This indicates that infections may not only be accompanied by the well-documented shift of zinc into the liver [29,30,31], but may also affect speciation of the remainder of serum zinc. In a rat model of surgical stress, changes in serum concentrations of total and free zinc could not be attributed to changes in albumin concentration alone [26]. Kelly et al. describe changes in zinc binding affinity for high and low molecular weight fraction proteins in the serum. They suggest a shift of zinc from the high molecular weight, high affinity fraction to low affinity or labile pools, consequently affecting the concentration of the different species in the serum [26].

**Figure 8:**
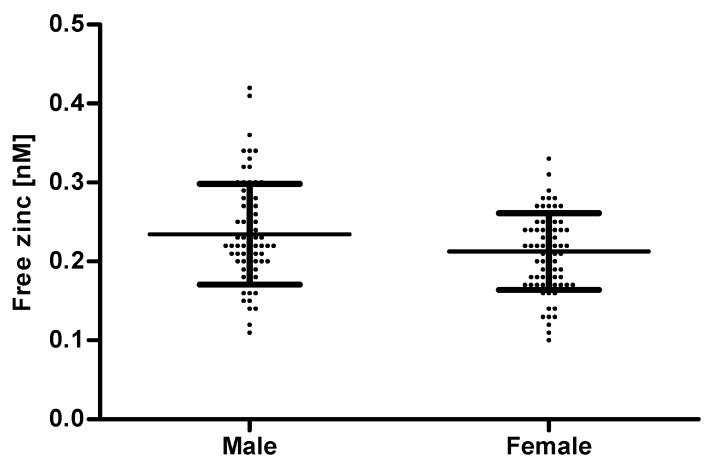
Comparison of the free zinc concentration in serum samples of males and females. The free zinc concentration was measured using the method established above. Data points represent means of n = 3 independent experiments and are depicted as scatter plots with means ± SD from 75 males and 79 females, respectively. Mean values for males and females were significantly different (unpaired t-test, p ≤ 0.05).

There was no correlation between free zinc and age (Supplemental Figure S1A). This seems surprising, as age is known to be correlated with a lower zinc status and even serum zinc levels decline with age [32,33]. However, in this case there was also no correlation between total serum zinc and age (Supplemental Figure S1B). This is probably due to the limited number of samples. A potential association between age and serum zinc should be analyzed in a larger cohort including individuals with age-related diseases. Comparing the free zinc concentration by gender, sera from females showed a significantly lower free zinc concentration (0.21 ± 0.05 nM) than those from males (0.23 ± 0.06 nM) (Figure 8). A similar difference between genders has also been seen by Bloxam et al. who detected a non-protein-bound zinc concentration of 24.5 ± 6 nM for the sera from females and 33.6 ± 3 nM for males [23].

Further correlation analyses with the total serum concentrations of the trace elements manganese, iron and selenium revealed no correlation of free serum zinc with any of these parameters. However, the serum concentration of copper was negatively correlated with the free zinc concentration (supplemental Table S1). As shown in supplemental Table S2, no difference between males and females has been observed for the serum concentrations of manganese, iron or selenium, but for copper a significantly higher concentration has been found in the sera from females (19.83 ± 0.64 nM) than in those from males (16.63 ± 0.23 nM), which is in line with previous reports [34,35]. The homeostases of zinc and copper are connected with regard to resorption and transport in the serum. High intake of zinc impairs copper resorption from lumen to blood [36]. Also, albumin functions as a transport protein for zinc as well as for copper. For copper and zinc the physiologically relevant binding sites are the N-terminal site (NTS) and the multi-metal binding site (MBS), respectively. However, MBS is also a secondary binding site for copper, although with a tenfold lower affinity than for zinc [37].

A quenching effect of copper on the fluorescent probe Zinpyr-1 has been described [38]. This could indicate that the significantly lower free zinc concentration in the sera of females compared to males might result from a disturbance of Zinpyr-1 by binding free copper, as there was more copper present in sera from females. However, this is confuted by two observations: first, the abovementioned study from Bloxam et al., which also reported lower free zinc in female subjects, was based on ultrafiltration, a method for which no interference between copper and zinc can be expected. Second, the negative correlation between free zinc and total copper concentrations in our study was limited to sera from females, while the sera of males do not even show a trend toward a negative correlation (supplemental Table S3). If serum copper would influence the fluorescent probe, reducing the determined free zinc concentration, the sera of males would have to show a similar effect as well.

Although several methods to determine free zinc or non-protein-bound zinc in serum samples have been described before [22,23,24,25,26,27], our assay offers several advantages. It is a 96-well plate format assay, allowing simultaneous quantification of multiple samples, making it suitable for high sample throughput. The equipment is more affordable and less extensive to maintain than, for example, an atomic absorption spectrometer used to determine the free zinc concentration after ultrafiltration of serum samples. It has been shown that the fluorescence intensities for F, F_min_ and F_max_ are relatively stable for a duration longer than the chosen incubation times, benefiting the robustness of the method. The sample volume required for this method is a particular advantage, as 10 µL of sample are sufficient for performing an analysis with three replicates, which is only a fraction of the requirement for the methods described in literature that we are aware of.

## 3. Materials and Methods

### 3.1. Materials

Ethylenediaminetetraacetic acid (EDTA) and 4-(2-hydroxyethyl)-1-piperazineethanesulfonic acid (HEPES) were obtained from Roth, Karlsruhe, Germany. Human AB serum (HS), N,N,N′,N′-tetrakis(2-pyridinylmethyl)-1,2-ethanediamine (TPEN) and ZnSO_4_ • 7 H_2_O were purchased from Sigma Aldrich, Steinheim, Germany. Zinpyr-1 was from Santa Cruz biotechnology, Dallas, USA, and Chelex^®^ 100 Resin from Bio-Rad, USA. All other chemicals were from standard sources.

### 3.2. Assay Principle

For quantification of the free zinc concentration with the low molecular weight fluorescent probe Zinpyr-1 the formula by Grynkiewicz et al. is applied [39]:

(1) $$\left[Zn^{2+}\right]=K_{D}\cdot \frac{F-F_{min}}{F_{max}-F}$$

Herein, K_D_ indicates the dissociation constant of the zinc:Zinpyr-1 complex of 0.7 nM [18], F_min_ the autofluorescence of the probe in the absence of zinc, F the fluorescence of the probe in the sample, and F_max_ represents the maximum fluorescence of the zinc-saturated probe. Notably, the dilution of the serum in the assay is not relevant for determining the free zinc concentration by this method, as outlined in detail in supplemental information SI 1.

In order to minimize the required sample volume, all fluorescence parameters were measured sequentially in the same well, starting with F, followed by the addition of a chelator to yield F_min_, and finally addition of excess zinc sulfate to reach F_max_. Incubations were carried out using a thermo-shaker (PST-60-HL4, Biosan, Riga, Latvia) at 37 °C and 250 rpm. Fluorescence measurements were performed at 37 °C at an excitation wavelength of 507 nm, emission wavelength of 526 nm, and bandwidths of 5 nm using a SPARK fluorescence plate reader (Tecan, Männedorf, Switzerland).

Assay-buffer (50 mM HEPES in bidistilled water adjusted to pH 6.5 with sodium hydroxide solution), was depleted of multivalent cations with Chelex^®^ 100 Resin according to the manufacturer’s protocol. This process increases the pH of the assay-buffer to a final value of 7.5. Measurements were performed in a volume of 100 µL of a 1:50 dilution in assay buffer in the wells of a 96-well-plate (Sarstedt, Nümbrecht, Germany). Samples were analyzed in triplicates, leading to a net sample requirement of 6 µL. Outer wells were not used for measurements, but filled with buffer, leaving a capacity of 60 wells, or 20 samples, per 96 well-plate.

Based on the results of the optimization outlined below the assay was finally performed with Zinpyr-1 (final concentration 0.05 µM) dissolved in 98 µL assay-buffer, equilibrated for 20 min before incubation was started by addition of 2 µL HS. Incubation times for F, F_min_, and F_max_ were 90, 20, and 90 min, respectively. For determination of F_min_ a final concentration of 100 µM of EDTA, and for F_max_ 500 µM zinc sulfate was applied. Serum samples were routinely stored at –21 °C. Results remained unchanged by five freeze/thaw cycles, indicating that this is without effect on the determined free zinc concentration (data not shown).

### 3.3. Optimization of Assay Parameters

To identify a potential impact of serum on excitation and emission wavelengths, zinc-saturated Zinpyr-1 in the absence or presence of HS was subjected to an excitation-scan from 400–526 nm with an emission wavelength of 545 nm and an emission-scan from 515–600 nm with an excitation wavelength of 495 nm (both in 2 nm steps, bandwidths 5 nm).

For F, F_min_ and F_max_ the incubation times as well as concentrations and, in the case of F_min_, the choice of chelator had to be optimized. First, fluorescence was recorded in 10 min intervals for 180 min after addition of HS in order to monitor the fluorescence for F. In a subsequent experiment, F_min_ was optimized by measuring F, followed by addition of 2 µL of either EDTA- or TPEN-solutions, measuring the fluorescence every minute for 60 min. To establish optimal conditions for F_max_, fluorescence intensity of F and F_min_ was measured, then 2 µL of different ZnSO_4_ stock solutions were added and fluorescence observed every 5 min for 180 min.

To determine the range of suitable concentrations of Zinpyr-1, the assay outlined above was performed ranging from 0.05 to 1 µM of the probe. Lower concentrations were not used to avoid limitations by instrument sensitivity.

### 3.4. Application of the Final Assay Procedure

The method was applied to a panel of 154 human serum samples. This study was conducted in adherence to the Declaration of Helsinki. The panel of human serum samples was obtained from a commercial supplier (in.vent Diagnostica GmbH, Hennigsdorf, Germany). The samples had been taken from self-reported healthy volunteers (75 male, 79 female) who provided their informed consent prior to sampling. In order to assess assay reliability, all samples were analyzed in three independent measurements (each in triplicates), leading to no remarkable differences. In order to ensure the reliability of the results on each plate a reference serum is carried along. The results of a run were considered reliable if the value of the reference serum was within the range of the mean value of the 32 total conducted runs ± 2 standard deviation (SD). The standard deviation of the reference serum for the 32 conducted runs was 19%.

### 3.5. Determination of Zinc, Iron, Copper, Manganese and Selenium by Inductively Coupled Plasma Mass Spectrometry (ICP-MS)

Zinc, iron, copper, manganese and selenium in commercial human serum samples were determined by an inductively coupled plasma mass spectrometry (ICP-MS/MS) system (Agilent ICP-QQQ-MS 8800, Waldbronn, Germany) using the method previously described in detail by Meyer et al. [40].

### 3.6. Statistical Analysis

Statistical significance of correlations was analyzed by Spearman rank correlation. After testing for normal distribution (D’Agostino and Pearson omnibus normality test) statistical significance of differences between means was analyzed by either unpaired t-test for normally distributed values or by Mann Whitney test for not normally distributed values, as indicated in the respective figures or tables. All calculations were performed using GraphPad Prism software version 5.01 (GraphPad Software Inc., CA, USA).

## 4. Conclusions

This assay is a fast high-throughput procedure to measure free serum zinc in a minimum amount of sample. It can be used in future studies to elucidate if the free zinc concentration in serum is an appropriate indicator for an individual’s zinc status, potentially even more suitable than total zinc in serum or plasma, as it reflects the bioavailable fraction. To this end, it has to be applied in larger-scale studies to evaluate its relationship to the body’s zinc status and look at alterations in response to zinc supplementation or undersupply. Furthermore, free zinc should be analyzed in different groups of patients with diseases for which an involvement of zinc has been shown, to see if the free zinc concentration might provide new insights in addition to total serum zinc. Other factors influencing the free zinc concentration in the serum will have to be explored along with possible connections with zinc homeostasis, to evaluate its suitability as a biomarker for an individual’s zinc status.

## Supplementary Materials

Supplementary materials can be found at https://www.mdpi.com/1422-0067/20/16/4006/s1.

## Abbreviations

ANOVA	analysis of variance
EDTA	Ethylenediaminetetraacetic acid
FA2	fatty acid binding site 2
HEPES	4-(2-hydroxyethyl)-1-piperazineethanesulfonic acid
HS	human AB serum
ICP-MS	inductively coupled plasma mass spectrometry
MBS	Multi-Metal Binding Site
NTS	N-terminal site
TPEN	N,N,N′,N′-tetrakis(2-pyridinylmethyl)-1,2-ethanediamine
