# Supplementary material for: A Zinpyr-1-based Fluorimetric Microassay for Free Zinc in Human Serum
Source: Int J Mol Sci. 2019 Aug 16;20(16):4006. doi: 10.3390/ijms20164006 (PMC6720863; doi:10.3390/ijms20164006)
Supplement: Supplementary file 1 [file ijms-20-04006-s001.pdf]

**Supplemental Material**

# A Zinpyr-1-based fluorimetric microassay for free zinc in human serum

Wiebke Alker[1,2], Tanja Schwerdtle[2,3], Lutz Schomburg[2,4], Hajo Haase [1,2,*]

[1] Department of Food Chemistry and Toxicology, Berlin Institute of Technology, Germany;
[2] TraceAge - DFG Research Unit on Interactions of essential trace elements in healthy and diseased elderly, Potsdam-Berlin-Jena, Germany
[3] Institute of Nutritional Science, University of Potsdam, Germany
[4] Institute for Experimental Endocrinology, Charité-Universitätsmedizin Berlin, Germany
* Correspondence: Haase@tu-berlin.de; Tel.: +49-30-314-727-01

## Abbrevations

ICP-MS, inductively coupled plasma mass spectrometry; HSA, human serum albumin; [HSA], concentration of zinc-free HSA; [ZnHSA], concentration of zinc-bound HAS, respectively of HSA-bound zinc; [$Zn^{2+}$], concentration of free zinc; [$Zn_{total}$], total zinc concentration; [$HSA_{total}$], total HSA concentration

**Supplemental Information**

Regarding the concentration of free zinc in a serum sample during the assay, the binding of zinc to human serum albumin can be described by the law of mass action (1):

$$(1) \qquad K_B = \frac{[ZnHSA]}{[Zn^{2+}]\cdot[HSA]}$$

This can be solved for free zinc $[Zn^{2+}]$ leading to (2):

$$(2) \qquad [Zn^{2+}] = \frac{[ZnHSA]}{[HSA]} \cdot \frac{1}{K_B}$$

Total zinc is the sum of free and albumin-bound zinc ($[Zn_{total}] = [Zn^{2+}] + [ZnHSA]$). This can be solved for albumin-bound zinc ($[ZnHSA] = [Zn_{total}] - [Zn^{2+}]$) and replaces the numerator in (2), leading to (3):

$$(3) \qquad [Zn^{2+}] = \frac{[Zn_{total}]-[Zn^{2+}]}{[HSA]} \cdot \frac{1}{K_B}$$

Likewise, total albumin is the sum of zinc-free and zinc-bound albumin ($[HSA_{total}] = [HSA] + [ZnHSA]$), which can be solved for zinc-free albumin ($[HSA] = [HSA_{total}] - [ZnHSA]$), and replaces the denominator in (3), resulting in (4):

$$(4) \qquad [Zn^{2+}] = \frac{[Zn_{total}]-[Zn^{2+}]}{[HSA_{total}]-[ZnHSA]} \cdot \frac{1}{K_B}$$

Moreover, under physiological conditions the concentration of serum albumin will always greatly exceed the total concentration of zinc ($[HSA_{total}] > [Zn_{total}]$). Hence, there is always sufficient albumin providing binding sites for zinc. As the equilibrium is strongly in favor of complex formation, the fraction of free zinc will be negligible compared to the amount of total zinc ($[Zn^{2+}] \ll [Zn_{total}]$), leading to the approximation that the term $[Zn_{total}]$ – $[Zn^{2+}]$ in (4) can be replaced by $[Zn_{total}]$, yielding (5):

$$(5) \qquad [Zn^{2+}] = \frac{[Zn_{total}]}{[HSA_{total}]-[ZnHSA]} \cdot \frac{1}{K_B}$$

Another consequence of virtually the entire total zinc existing in its albumin-bound form ($[ZnHSA] \sim [Zn_{total}]$) allows the approximation of replacing the concentration of albumin-bound zinc $[ZnHSA]$ in (5) by the total concentration of zinc $[Zn_{total}]$, leading to (6):

$$(6) \qquad [Zn^{2+}] = \frac{[Zn_{total}]}{[HSA_{total}]-[Zn_{total}]} \cdot \frac{1}{K_B}$$

From (6) it can be derived that the concentration of free zinc in the serum sample is dependent on the ratio between $[Zn_{total}]$ and $[HSA_{total}]$ as well as the binding constant. Therefore, dilution of the serum sample in the well of the 96-well plate with assay-buffer does not have to be taken into account when calculating the free zinc concentration.

**SI 1: Calculation of the dissociation equilibrium in the established assay between zinc and human serum albumin.**

## Supplemental Figures

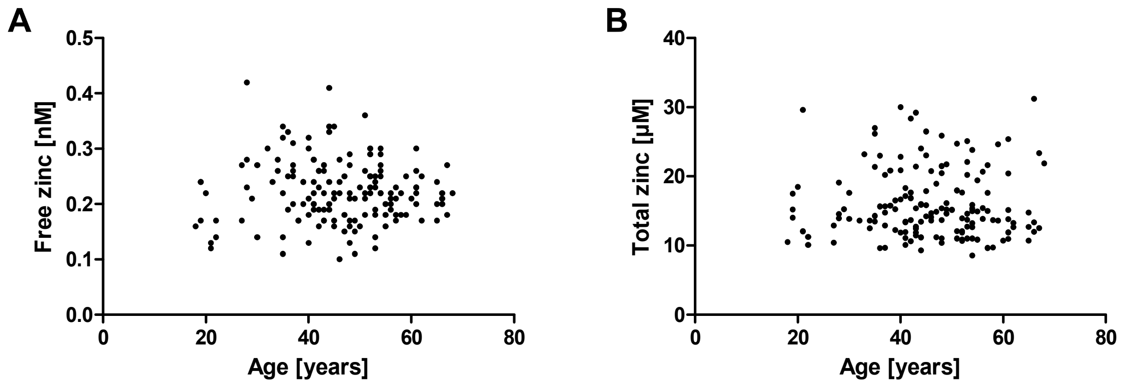


**Figure SF1: Correlation between free and total zinc concentrations and age of the donors.** In sera of 154 patients aged between 18 and 68 years, the free zinc concentration was measured using Zinpyr-1 (A) and total zinc by ICP-MS (B). No significant correlation with age has been found for either value (Spearman rank correlation, $\alpha = 0.05$).

## Supplemental Tables

**Table ST1: Correlation between concentration of free zinc with total zinc, manganese, iron, copper, and selenium.**

| | Zn | Mn | Fe | Cu | Se |
|---|---|---|---|---|---|
| **Spearman r** | –0.02549 | –0.1116 | –0.01660 | –0.2131 | –0.01969 |
| **P value (two-tailed)** | 0.7536 | 0.1908 | 0.8381 | 0.0080 | 0.8085 |
| **P value summary** | ns | ns | ns | ** | ns |
| **Is the correlation significant? (alpha=0.05)** | No | No | No | Yes | No |

The free zinc concentration was measured using the established method, the concentration of total zinc, manganese, iron, copper and selenium by ICP-MS. (ns, not significant; ** p ≤ 0.01, Spearman rank correlation)

**Table ST2: Differences between the serum samples of males and females in total zinc, manganese, iron, copper or selenium concentration.**

| | Zn male/ female | Mn male/ female | Fe male/ female | Cu male/ female | Se male/ female |
|---|---|---|---|---|---|
| **Unpaired *t*-test P value** | 0.5199 | 0.5096 | 0.4899 | < 0.0001 | 0.4435 |
| **P value summary** | ns | ns | ns | *** | ns |
| **Are means significantly different? (P < 0.05)** | no | no | no | yes | no |

The concentration of total zinc, manganese, iron, copper and selenium was measured by ICP-MS. (ns, not significant; ***p ≤ 0.001, Mann–Whitney test)

**Table ST3: Correlation between concentration of free zinc and total copper in the serum samples of females and males.**

| | Cu female | Cu male |
|---|---|---|
| **Spearman r** | –0.3339 | 0.02316 |
| **P value (two-tailed)** | 0.0026 | 0.8437 |
| **P value summary** | ** | ns |
| **Is the correlation significant? (alpha=0.05)** | Yes | No |

The free zinc concentration was measured using the established method, the concentration of total copper by ICP-MS. (ns, not significant; ** p ≤ 0.01, Spearman rank correlation)
